# Neutrophil-derived microparticles induce myeloperoxidase-mediated damage of vascular endothelial cells

**DOI:** 10.1186/1471-2121-15-21

**Published:** 2014-06-11

**Authors:** Thassila Nogueira Pitanga, Luciana de Aragão França, Viviane Costa Junqueira Rocha, Thayna Meirelles, Valéria Matos Borges, Marilda Souza Gonçalves, Lain Carlos Pontes-de-Carvalho, Alberto Augusto Noronha-Dutra, Washington Luis Conrado dos-Santos

**Affiliations:** 1 Fundação Oswaldo Cruz-BA, Gonçalo Moniz Research Center, Salvador, BA, Brazil; 2 Health Science Institute, Federal University of Bahia, Salvador, BA, Brazil; 3 Heart Institute, Faculty of Medicine, USP, São Paulo, SP, Brazil; 4 University College London, Gower Street, London, UK

**Keywords:** Neutrophil microparticles, Endothelial cell injury, Myeloperoxidase, Oxidative stress

## Abstract

**Background:**

Upon activation neutrophil releases microparticles - small plasma membrane vesicles that contain cell surface proteins and cytoplasmic matter, with biological activities. In this study we investigated the potential role of myeloperoxidase in the endothelial cell injury caused by neutrophil-derived microparticles.

**Results:**

Microparticles were produced by activating human neutrophils with a calcium ionophore and characterized by flow cytometry and transmission and scanning electron microscopy. Myeloperoxidase activity was measured by luminol-dependent chemiluminescence. Neutrophil microparticles-induced injuries and morphological alterations in human umbilical vein endothelial cells (HUVECs) were evaluated by microscopy and flow cytometry. Neutrophil microparticles were characterized as structures bounded by lipid bilayers and were less than 1 μm in diameter. The microparticles also expressed CD66b, CD62L and myeloperoxidase, which are all commonly expressed on the surface of neutrophils, as well as exposition of phosphatidylserine. The activity of the myeloperoxidase present on the microparticles was confirmed by hypochlorous acid detection. This compound is only catalyzed by myeloperoxidase in the presence of hydrogen peroxide and chloride ion. The addition of sodium azide or taurine inhibited and reduced enzymatic activity, respectively. Exposure of HUVEC to neutrophil microparticles induced a loss of cell membrane integrity and morphological changes. The addition of sodium azide or myeloperoxidase-specific inhibitor-I consistently reduced the injury to the endothelial cells. Taurine addition reduced HUVEC morphological changes.

**Conclusions:**

We have demonstrated the presence of active myeloperoxidase in neutrophil microparticles and that the microparticle-associated myeloperoxidase cause injury to endothelial cells. Hence, the microparticle-associated myeloperoxidase-hydrogen peroxide-chloride system may contribute to widespread endothelial cell damage in conditions of neutrophil activation as observed in vasculitis and sepsis.

## Background

Neutrophil activation by anti-neutrophil cytoplasmic antibody or complement plays a central role in the genesis of a variety of small vessels vasculitis. These conditions are associated with significant morbidity and mortality due to lesions in central nervous system, lungs and kidneys. Although direct neutrophil contact and granule release are recognized mechanism of endothelial cell aggression by neutrophils, evidence suggests that activated neutrophils also release microparticles (MPs) that may contribute to tissue lesion in the course of vasculitis [1,2].

Microparticles are vesicles smaller than 1 μm in diameter that are released from plasma membranes of several cell types after cell activation or apoptosis in response to various stimuli, including lipopolysaccharide (LPS), formyl-methionyl-leucyl phenylalanine (fMLF), phorbol 12-myristate 13-acetate (PMA) and calcium ionophore [3-6]. MPs have been implicated in inflammation, coagulation and endothelial dysfunction. Circulating levels of MPs are augmented in several inflammatory diseases including vasculitis [3,7].

Neutrophil MPs contain inflammatory mediators such as 5-leukotrien platelet activating factor and adhesion molecules including CD11a, CD11b and selectins [3,8]. A few studies have provided evidence that human neutrophil MPs contain active myeloperoxidase (MPO), suggesting that they may activate endothelial cells [1,8]. This condition may also give raise to lesions present in some vasculitis [3]. Recently, Hong and colleagues (2012) showed that neutrophils primed with TNFα and stimulated with ant-neutrophil cytoplasmic antibodies (ANCA) antibodies release microparticles that activate endothelial cells by a reactive oxigen species (ROS)-dependent mechanism [1]. However, little is known about the pathways of potential endothelial injury involving neutrophil MPs and ROS.

MPO, a heme protein found predominantly in neutrophils, is synthesized and stored in azurophilic granules of granulocytes and monocytes [9-11]. The key feature of this enzyme is its ability to catalyze the oxidation of chloride ions to hypochlorous acid through hydrogen peroxide reduction at neutral pH and physiological plasma concentrations of halide [11,12]. HOCl is a ROS with potent microbicidal and viricidal activities and is thought to play an essential role in normal leukocyte function [13]. However, HOCl also has the capacity to damage host tissue [9,11-13]. This oxidant causes endothelial dysfunction [11] and caspase activation that results in apoptosis of human endothelial cells [14,15]. Evidence suggest that it also participates in lipoprotein oxidation [3] and development of atheroma [14].

In this study we investigated the potential role of MPO in endothelial cell injury caused by neutrophil MPs. In order to do that we isolated and characterized MPs produced by activated neutrophils and incubated these MPs with cultivated human umbilical vein endothelial cells (HUVEC) in presence of hydrogen peroxide and chloride ion. The data presented here offer evidence that the neutrophil MPs-associated myeloperoxidase-hydrogen peroxide-chloride pathway may contribute to endothelial cell lesion and consequently to expand the endothelial cell damage observed in conditions of neutrophil activation such as vasculitis and sepsis.

## Methods

### Isolation of human granulocytes

Neutrophils were obtained from heparinized blood of healthy human donors according to a previously described technique [16]. Approximately 25 mL of venous blood was gently mixed with 10 mL of 3% Dextran T500 (Sigma, St Louis, MO, USA) and left for 25 min for erythrocyte sedimentation. The leukocyte-rich supernatant was aspirated and centrifuged at 750 g for 10 min at 25°C. The pellet was resuspended in 6 mL of Hank’s Balanced Salt Solution (HBSS) without Ca^2+^, Mg^2+^ or phenol red. This cell suspension was layered over 3 mL of Histopaque-1077 (Sigma) and centrifuged at 800 g for 20 min at 25°C. After removing the supernatant, the polymorphonuclear neutrophils pellet was resuspended in 2 mL of phosphate-buffered saline (PBS), and 5 mL of ultra-pure water was added to hypotonically lyse the residual erythrocytes. After 1 min, isotonicity was restored by adding 2 mL of 3.5% NaCl. The cells were then centrifuged at 150 g for 10 min at 4°C and resuspended in 10 mL of HBSS with Ca^2+^/Mg^2+^. The purity of the granulocyte suspension accessed by flow cytometry using anti-CD66b labeling (a granulocyte activation marker [17]) was higher than 90%. The experimental protocol was approved by the Ethics Committee on Human Research, Gonçalo Moniz Research Center, Salvador, Brazil (protocol No. 313, CAAE 0024.0.225.000-09).

### MP isolation and protein content determination

The isolation of MPs was performed as previously described [16,18]. Neutrophils were stimulated with 2 μM calcium ionophore (A23187) (Sigma) for 20 min followed by centrifugation at 8000 g for 6 min at 4°C. The upper MPs-containing supernatant was collected and spun down at 100,000 g for 45 min at 4°C. MPs present in the sediment were washed once with HBSS without phenol red, resuspended in 500 μL of PBS and stored at 4°C until use. The protein concentrations of the MPs suspensions were measured using the BCA method (Thermo Scientific, Waltham, MA, USA) according to the manufacturer’s protocol.

In order to evaluate if other other stimuli also generated similar MPs, neutrophils were activated with 2 μM calcium ionophore (A23187) or 200 nM phorbol-12-myristate-13-acetate (PMA, Sigma) for 30 min at 37°C, or with 10 μg/mL lipopolysaccharide (LPS; Sigma) for 2 hours at 37°C, or were irradiated with ultraviolet light for 20 min and incubated for 1 h at 37°C. The MPs were then isolated and quantified as described above.

### Flow cytometry analysis of MPs

Microparticles surface membranes were labeled with PKH26 (red fluorescence; Sigma) following the labeling procedure provided by the manufacturer. Briefly, 100 μL of a 1 μg/mL MPs suspension was incubated with 100 μL PKH26 (1 μM) solution for 1 min at 25°C. RPMI media 1640 (200 μL; Sigma) supplemented with 10% fetal bovine serum (Gibco, Carlsbad, CA, USA) was added to stop the reaction. Labeled MPs were separated from the remaining dye and washed in PBS by ultracentrifugation at 100,000 g for 45 min. MPs were resuspended in 300 μL HBSS and analyzed by flow cytometry in the red fluorescence channel (FL2).

Neutrophils (2 × 10^7^/mL) were labeled with 20 μM carboxyfluorescein diacetate (CFDA; Sigma) for selective tagging of cytoplasmic contents and then stimulated with 2 μM calcium ionophore (A23187) to confirm that the MPs retained cytoplasmic contents and the integrity of their membranes. MPs derived from labeled neutrophils were obtained and separated from the remaining dye, washed with PBS by ultracentrifugation at 100,000 g for 45 min and resuspended in HBSS.

Microparticles were studied in the presence of flow count fluorescent calibrator beads (1 μm diameter; Invitrogen, Carlsbad, CA, USA). Those beads were run to calibrate the size and distribution of MPs. MPs gate was defined as the events lower than 1 μm diameter on the forward scatter (FSC) and side scatter (SSC) parameters plotted on logarithmic scales. In addition, MPs were labeled with several fluorochrome-conjugated antibodies: anti-CD66b-FITC (BD Pharmingen, San Jose, CA, USA), anti-CD62L (BD Bioscience, San Jose, CA, USA), anti-MPO-PE (Novus Biologicals, Littleton, CO, USA) and control isotypes (AbD Serotec, Raleigh, NC, USA). The MPs were resuspended in 300 μL HBSS with or without 1 mM CaCl_2_, and 0,5 μg/mL Annexin V-PE (AnV, BD Bioscience) was added to each reaction. A minimum of 100,000 events per sample were collected using CellQuest software (BD Bioscience). The experiments were analyzed using Flow Jo software (Tree Star Inc., Ashland, OR, USA).

### Transmission electron (TEM) and scanning electron (SEM) microscopies

Microparticles were analyzed by TEM using a previously described technique [16]. MP-containing pellets were fixed with 2% glutaraldehyde (Sigma) in PBS and post-fixed with 2% osmium tetroxide (Sigma). The samples were embedded in araldite resin. Ultra-thin sections were stained with 2% uranyl acetate in 50% ethanol followed by Reynolds lead citrate (Polysciences Inc., Warrington, PA, USA). The sections were analyzed under TEM (ZEISS EM109).

For SEM, MPs were fixed in Karnovsky fixative, dehydrated in alcohol and stored on a glass surface for 20 min at room temperature. The glass surface was metallized with gold and observed under a scanning microscope (JSM 6390 LV Low Vacuum; JEOL, Peabody, MA, USA). Images were obtained by secondary electron analysis at a working distance of 15 to 25 mm and an accelerating voltage of 20 to 25 kV.

### Luminol-dependent chemiluminescence assay

Myeloperoxidase activity was assessed by estimating HOCl production using a previously described chemiluminescence (CL) method [19]. Briefly, 1 mL of MP suspension (1 μg/mL) was plated on a 35-mm dish and used for CL measurement in a photon-counting device composed of a gallium arsenide photomultiplier tube (Hamamatsu R943) thermoelectrically cooled to -20°C. Samples were placed in dishes, sealed with cling film and maintained at 37°C in a thermostatic light-sealed chamber. Their CL emissions were then collected by reflections off a concave mirror and focused onto the photomultiplier tube. HOCl production derived for myeloperoxidase-hydrogen peroxide-chloride system (MPO/H_2_O_2_/Cl^-^ system) was recorded by luminescence after the addition of 20 μM luminol to the culture for 20 min. The HOCl and luminol interaction-derived CL was measured in intact MPs using HBSS plus 10 mM HEPES (pH 7.3) and 20 μM H_2_O_2_. The HOCl measurement was further conducted in the presence of sodium azide (NaN_3_; 10 μM) and taurine (1 mM; Sigma). To confirm the luminescence dependency upon chloride, samples of MPs and luminol were also prepared with HBSS without chlorine. Previously to analyses, baseline readings were performed, and CL emission do not exceed 17 counts/seconds.

### Human umbilical vein endothelial cell culture

HUVEC were isolated from fresh umbilical cords after treatment with collagenase (0.1% - Worthington Diagnostic systems Inc., Freehold, NJ, USA) using a previously described technique [8] with modifications. HUVECs were cultured in RPMI-1640 medium supplemented with 10% fetal bovine serum, antibiotic (50 μg/mL gentamicin) and 100 μg/mL endothelial cell growth supplement (BD Biosciences) and used at passages 4 to 6. The cells were grown in 5% CO_2_ at 37°C, and the medium was replaced every 2 days.

### Assessment of HUVEC plasma membrane integrity

HUVEC were incubated with MPs and H_2_O_2_ for 30 min in 5% CO_2_ at 37°C and then subjected to double labeling with 2.4 μM propidium iodide (PI; Sigma) and 20 μM CFDA for 10 min, to assess plasma membrane integrity. PI (red fluorescent probe) has affinity for DNA and only permeates cells with non-intact membranes. CFDA, in contrast, permeates intact cell membranes and is quickly converted by intracellular esterases into a green fluorescent derivative [20]. Two inhibitors, sodium azide and Myeloperoxidase Inhibitor-I (a benzoic acid hydrazide analog; Merck KGaA, Darmstadt, Germany), were used in this experiment. The cells were washed with HBSS, incubated with 1.7 mM formaldehyde for 8 min and then washed again. Next, 7 μL of Prolong Gold reagent (Invitrogen) containing 4,6′-diamino-2-phenylindole (DAPI; Invitrogen) was added to each culture. The cultures were visualized using a fluorescence microscope (Olympus BX51) and analyzed with an Image Pro-Plus analysis system. The injury was quantified by percentage of cells stained with PI per section for groups with or without peroxidase inhibitor.

Additionally, after being treated with MPs, 20 μM H_2_O_2_, 10 μM NaN_3_ and 1 mM taurine, for 30 min in 5% CO_2_ at 37°C, the HUVEC were double labeled with AnV and PI and analyzed by flow cytometry to assess plasma membrane damage. The HUVEC were resuspended in 300 μL HBSS with or without 1 mM CaCl_2_, 0,5 μg/mL AnV and 2 μg/mL PI. Data from a minimum of 10,000 events per sample were collected using CellQuest software (BD Bioscience). The experiments were analyzed using the Flow Jo software (Tree Star Inc., Ashland, OR, USA).

### Determination of HUVEC morphology

HUVEC were grown in 35 × 10 mm plates (5 × 10^5^ cells/dish) and maintained at 37°C in 5% CO_2_. After 3 days, the cells were treated with 5 μg/mL MPs in HBSS containing 20 μM H_2_O_2_ with or without 1 mM taurine. The cells were cultured with the MPs for 2 and 16 min and photographed at the end of each period. As a control, endothelial cells were maintained during the same time intervals with HBSS alone or with 20 μM H_2_O_2._ Endothelial cell images were captured using a phase contrast microscope (Olympus CK2, Center Valley, PA, USA), and the images were analyzed using Image Pro Plus 6.1 software (Media Cybernetics, Rockville, MD, USA).

### Statistical analysis

All results were expressed as median with interquartile range. Comparisons between groups treated or untreated with inhibitors were made by Mann–Whitney *U* test. Differences with P < 0.05 were considered significant.

## Results

### Characteristics of calcium ionophore-induced neutrophil MPs

**Figure 1:**
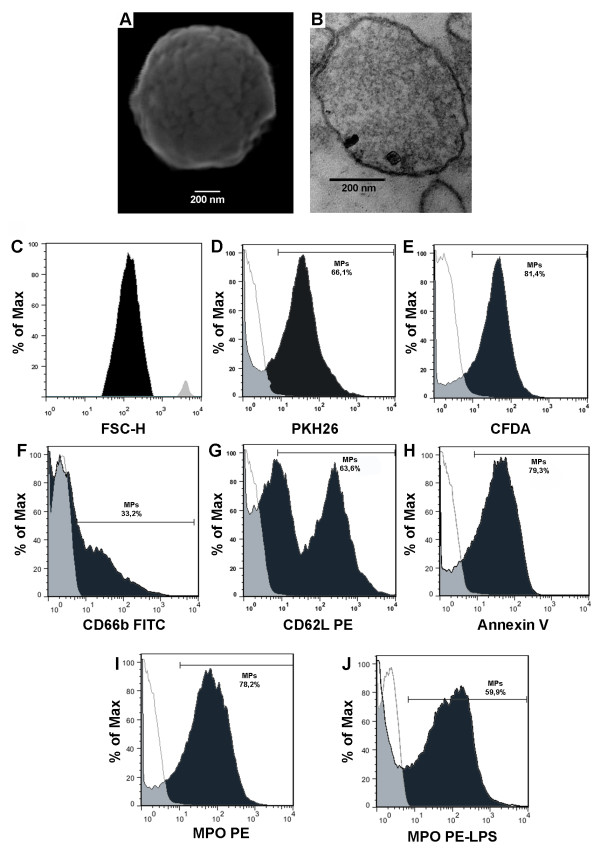
**Characterization of neutrophil microparticles (MPs).** Neutrophil MPs analyzed by electron microscopy **(A-B)** and flow cytometry **(C-I)**: Representative scanning electron microscopy micrograph of spheroid MPs **(A)**. MPs analyzed by transmission electron microscopy and show the presence of lipid bilayers **(B)**. Fluorescence histogram of MPs (black) and beads (grey), demonstrating their size **(C)**. MPs events were marked with 2 μM PKH26 a membrane marker (black) **(D)**. Integrity of neutrophil MPs **(E)**: Fluorescence histogram of MPs derived from neutrophils labeled with 20 μM CFDA (black) and activated with 2 μM calcium ionophore. Flow cytometry analysis of the surface protein expression of MPs derived from neutrophils activated with 2 μM calcium ionophore **(F-I)**: MPs were incubated with isotype control antibodies (thick line) or specific antibodies (black). Polymorphonuclear degranulation marker CD66b **(F)**. L-selectin (CD62L) **(G)**. Phosphatidylserine **(H)**: MPs were labeled with annexin V (AnV) in the presence (black) or absence (negative control- thick line) of calcium. Myeloperoxidase **(I)**. MPs derived from neutrophils activated with LPS: events were labeled with anti-Myeloperoxidase (black) **(J)**. The figure shows a representative experiment among three performed.

MPs were isolated by differential centrifugation of neutrophils activated with 2 μM calcium ionophore and characterized by electron microscopy and flow cytometry (Figure 1). Representative SEM micrographs revealed spheroid MPs smaller than 1 μm and demonstrated that the preparations did not contain impurities (Figure 1A). TEM micrographs showed the presence of lipid bilayers in MPs (Figure 1B). The size of the MPs was confirmed by flow cytometry using 1-μm-diameter fluorescent microbeads as a reference (Figure 1C). PKH26 incorporation confirmed the lipid nature of the membranes associated with MPs (Figure 1D).To assess the membrane integrity and presence of cytoplasmic contents, MPs were stained with CFDA and analyzed by flow cytometry. Approximately 80% of the total particles with less than 1 μm diameter exhibited positive labeling for CFDA, corresponding to intact MPs containing cytoplasmic material (Figure 1E).Under normal circumstances, MPs released by activated neutrophil express molecules characteristic of this cell type. Hence, we analyzed protein expression on the surface of MPs using mAbs against different neutrophil proteins. The MPs were 33,2% CD66b positive (Figure 1F), 63,6% CD62L positive (Figure 1G), 79,3% of MPs were AnV positive, indicating the presence of PS as expected (Figure 1H). Myeloperoxidase was detected associated with MPs, regardless of the type of stimulus, being present in MPs released by neutrophils activated by calcium ionophore (A23187) (78.2% Figure 1I) or LPS (59.9%, Figure 1J).

### Catalytic activity of the MPs-associated MPO

**Figure 2:**
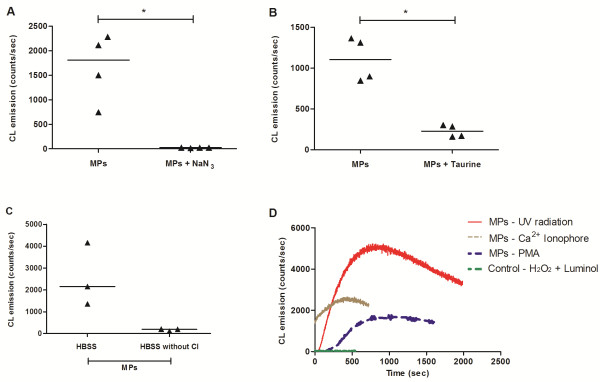
**Myeloperoxidase activity in neutrophil MPs measured by chemiluminescence (CL).** Effect on luminol CL of MPs (1 μg/mL) and 20 μM H_2_O_2_ with/without 10 μM sodium azide (NaN_3_) (n = 4) **(A)**; with/without 1 mM taurine (n = 4) **(B)** and with/without Cl (n = 3) **(C)**. MPO activity was observed on MPs derived from neutrophils activated with different stimulus (irradiation with ultraviolet, calcium ionophore and PMA) **(D)**. Data represent the median with interquartile range of three or four independent experiments (*p < 0.05, Mann Whitney test).

MPO activity was measured using a HOCl-induced luminol CL system. As shown in Figure 2, the luminol solution alone (HBSS, luminol and H_2_O_2_) emitted no luminescence. The addition of MPs into the 20 μM luminol solution led to a high level of photon emission. Sodium azide, an inhibitor of peroxidase, decreased the luminescence to basal levels (Figure 2A, p < 0.05). The addition of taurine (1 mM), a HOCl scavenger, also decreased the luminescence (Figure 2B, p < 0.05), although it showed a less pronounced effect than sodium azide. Furthermore, incubating MPs in a medium deprived of chlorine resulted in a very low luminescence emission, suggesting the HOCl production by CL reaction (Figure 2C). Therefore, the luminescence emitted indicates that neutrophils MPs possess active MPO. To evaluate whether different stimulus were also capable of generating MPs with active MPO, neutrophils were activated with 200 nM PMA or irradiated with ultraviolet (Figure 2D). Although differences were observed in luminescence intensity, both stimuli generated MPs with catalytically active MPO.

### Exposure of HUVECs to the MPs/H_2_O_2_/Cl^-^ system

**Figure 3:**
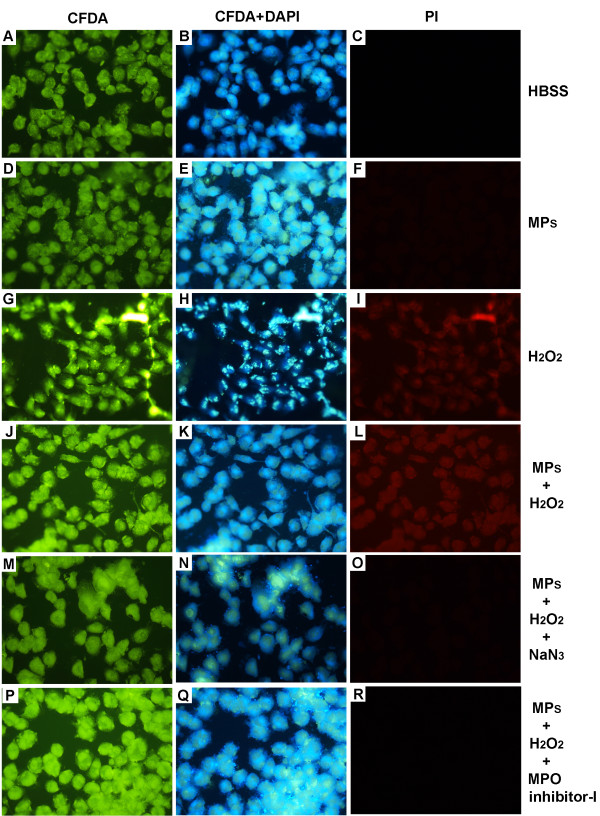
**Loss of integrity of cell membrane of HUVEC after exposure to neutrophil MPs and H**_**2**_**O**_**2**_**.** CFDA-labeled cells **(A, D, G, J, M and P)**; PI-labeled cells **(B, E, H, K, N and Q)**; merged figures **(C, F, I, L, O and R)**. Control HUVECs incubated with medium alone **(A-C)**. HUVECs incubated with 5 μg/mL MPs **(D-F)**. HUVECs incubated with 20 μM H_2_O_2_**(G-I)**. HUVECs incubated with 5 μg/mL MPs and 20 μM H_2_O_2_**(J-L)**. HUVECs incubated with 5 μg/mL MPs, 20 μM H_2_O_2_ and 10 μM sodium azide **(M-O)**. HUVECs incubated with 5 μg/mL MPs, 20 μM H_2_O_2_ and 1 μg/mL MPO inhibitor-I **(P-R)**. Magnification: 400×. The results shown in each panel are representative of four independent experiments.

Once the activity of MPO of neutrophil MPs was detected, we investigated whether this enzyme could mediate the damage caused by MPs to endothelial cells. The cells were incubated for 30 min with HBSS alone or containing an amount of MPs that corresponded to 5 μg of protein per mL and 20 μM H_2_O_2_ with or without inhibitors. HUVECs incubated with HBSS or MPs alone did not exhibit changes in membrane integrity (Figure 3A-C). On the other hand, MPs/H_2_O_2_/Cl^-^ system induced cytotoxicity, revealed by positive staining for PI, which indicates loss of lipid bilayer integrity.

**Figure 4:**
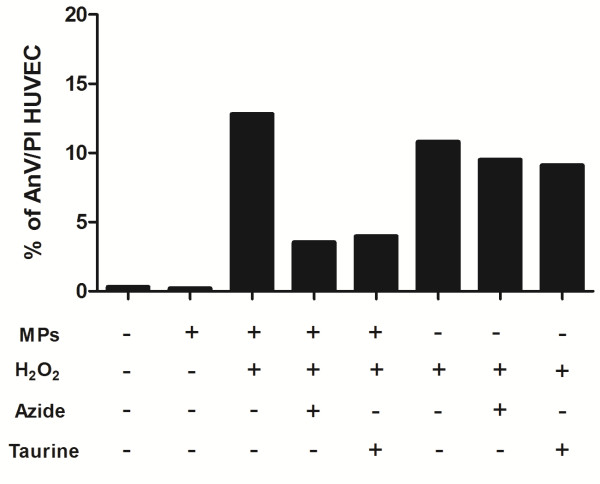
**Neutrophil MPs induce cell injury by MPO activity.** HUVEC cells were incubed with neutrophil MPs (5 μg/mL) in the presence or absence of 20 μM H_2_O_2_, 10 μM sodium azide and 1 mM taurine, for 30 min in 5% CO_2_ at 37°C. After the treatments, the cells were submitted to AnV and PI labeling and analyzed by flow cytometry. The injury caused by the MPO system (MPs and H_2_O_2_) and not the injury induced by H_2_O_2_ alone was decreased in the presence of sodium azide or taurine, inhibitors of the MPO pathway. The values represent the percent of AnV and PI positives cells in each treatment in relation to the total cells analyzed.

**Figure 5:**
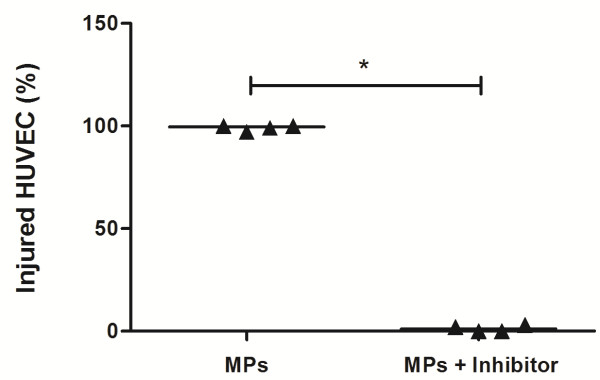
**Percentage of injured HUVECs measured after exposure to neutrophil MPs and H**_**2**_**O**_**2**_**.** PI-labeled HUVEC (representing injured cells) were enumerated with a fluorescence microscope. HUVEC were observed after the addition of 5 μg/mL MPs and 20 μM H_2_O_2_ with/without inhibitor (sodium azide or MPO inhibitor-I). Both inhibitors were similarly effective in protecting against cell injury. Data represent the median with interquartile range of four independent experiments. (*P < 0.05, Mann Whitney test).

To determine whether the cytotoxic effect of the MPs depended on MPO activity, we subjected HUVEC to different treatments, stained them with PI and examined by fluorescence microscopy, or stained with PI and AnV and examined by flow cytometry. HUVEC incubation with HBSS (Figure 3A-C and Figure 4) or MPs alone (Figure 3D-F and Figure 4) did not produce endothelial cell injury. As expected, treatment with H_2_O_2_ alone (Figure 3G-I, and Figure 4) or with the MPs/H_2_O_2_/Cl^-^ system (Figure 3J-L and Figure 4) produced cell damage. However, the HUVEC injury produced by the MPs/H_2_O_2_/Cl^-^ system but not the injury produced by H_2_O_2_ alone is significantly decreased in the presence of reagents that interfere with the MPO pathways such as 10 μM NaN_3_ (Figure 3M-O and Figure 4), 1 μg/mL MPO Inhibitor-I (Figure 3P-R) or taurine (Figure 4 and Figure 5). These data show that the endothelial cell damage induced by neutrophil MPs is dependent on MPO, while the damage induced by H_2_O_2_ alone is not associated with this pathway of cell damage.

**Figure 6:**
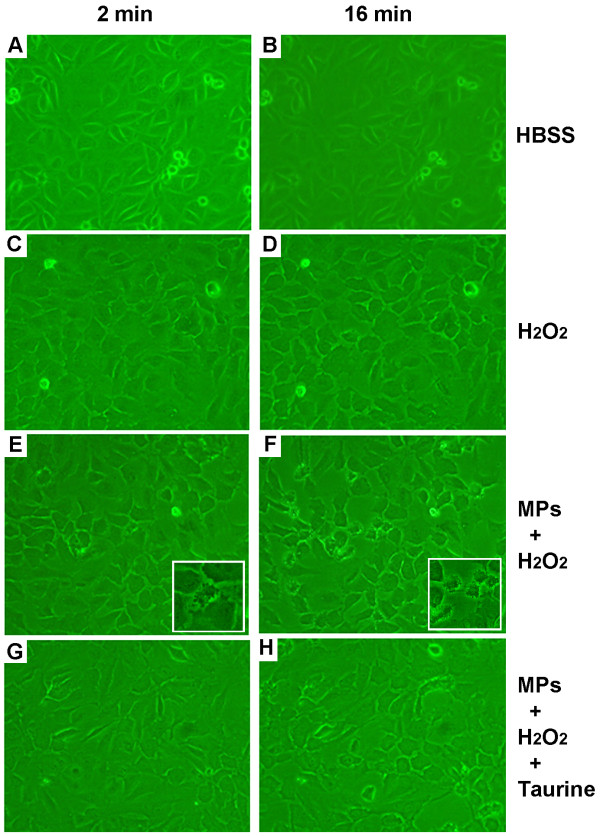
**Morphological changes in HUVEC after exposure to neutrophil MPs and H**_**2**_**O**_**2**_**.** HUVEC incubated for 2 min **(A)** and 16 min **(B)** with HBSS. Cells were observed with 2 min **(C)** and 16 min **(D)** after the addition of H_2_O_2_. Note the increased blebbing cells observed with 2 min, **(E)** and 16 min **(F)** after the addition of 5 μg/mL MPs and 20 μM H_2_O_2_ (inserts) indicating cell damage. Cells treated with 1 mM taurine showed a lower number of morphological changes with 2 min **(G)** and 16 min **(H)**. Magnification: 100×. The figures show a representative experiment among four performed.

Cytotoxicity was also evaluated by phase contrast optical microscopy in endothelial cell cultures. As expected, compared to negative control (Figure 6A, B), H_2_O_2_ alone induced some modification on the shape of the endothelial cells surface after 2 and 16 min of incubation (Figure 6C, D). However, in presence of MPs/H_2_O_2_/Cl^-^ system, blebs were formed on the surface of the HUVEC in a time-dependent manner (“popcorn-like” aspect) (Figure 6E, F), as shown in the inserts highlighting the blebbing cells. The inhibition of membrane disruption or surface blebs formation by treatment with MPO inhibitors or taurine confirmed that HOCl was involved in the process (Figure 6G, H).

## Discussion

In the present study, we isolated and characterized MPs derived from human activated neutrophils and showed that these MPs are capable of causing injury to vascular endothelial cells in an MPO-dependent manner.

We obtained MPs derived from neutrophils activated with calcium ionophore A23187. This method is widely used to induce the release of MPs *in vitro*[18]. Although neutrophil MPs were shown to differ in their composition and functional properties depending on the stimulating agent [4,5,8,10], here we show that the MPs produced using calcium ionophore present similar characteristics to those produced by neutrophils incubation with ANCA and other inflammatory stimuli [1]. For instance, these MPs appeared on TEM, SEM and flow-cytometry analysis as round vesicles of heterogeneous size, enclosed by lipid bilayers and measuring less than 1 μm, in accordance with published data [3-5,8,11,21]. The membrane integrity of these vesicles was maintained as shown by flow cytometry using CFDA labeled, a marker that permeates intact cell and is quickly converted by cytoplasmic esterases into a green fluorescent derivative [19]. As reported in the literature, the neutrophil MPs used in this study expressed PS, L-selectin (CD62L) and CD66b. CD66b is also widely used to define that the MPs originate from activated neutrophils [5,8,10,12,22].

Both CD62L and CD66b are adhesion molecules expressed on activated granulocytes that are involved in their adherence to endothelial cells and leukocyte migration into tissues [3-5,8,11,21]. The expression of these molecules on the surface of MPs may allow their adherence to endothelial cells.

In this study we did not examine the actual process of MPO release to the endothelial cells. However, the MPO staining using specific antibody in flow cytometry was achieved by using intact microparticles that were non-permeable to macromolecules. This fact suggests that the cell damage is caused by the product of the reaction that occurs in the vesicular surface. Further studies are needed in order to confirm this hypothesis.

We demonstrated by CL that the quantity of counts/seconds detected in preparations containing MPs, H_2_O_2_ and Cl^-^ ions was approximately 80-fold higher in complete HBSS than in preparations with modified HBSS (without chlorine). Because the extracellular luminescence of stimulated neutrophils can be completely suppressed by MPO inhibitors or partially inhibited by HOCl scavengers of [13], the complete inhibition of CL in MPs exposed to the MPO inhibitor NaN_3_[13] supports the hypothesis that the luminol CL is associated with the myeloperoxidase-catalyzed generation of HOCl. No significant luminescence was detected in absence of MPs or using chloride-free medium. Similarly, CL emission was reduced by taurine, a HOCl scavenger [15]. These findings confirm the presence of catalytically active MPO associated with the neutrophil vesicles in this study and strongly suggest that HOCl is the agent that mediates this reaction.

We also note that, despite the association of several molecules in MPs to be dependent on the stimulus used [9], the MPO was present in MPs derived from activated neutrophils with ionophore calcium A23187 or LPS (a pro-inflammatory lipid mediator), demonstrating that the presence of MPO in MPs independent of inductive stimulus of blistering. The presence of MPO surface of MPs was demonstrated by MPs derived from activated neutrophils with fMLP [9].

Culturing HUVEC in the presence of neutrophil MPs in medium containing H_2_O_2_ and chlorine provided evidence for HOCl-induced toxicity on these cells. Previous studies have shown that low concentrations of purified HOCl induce apoptosis in HUVEC, whereas high concentrations induce necrosis [14,15].

Neutrophil MPs damaged endothelial cells within a short period of time. We observed a loss of membrane integrity in endothelial cells after 30 minutes of incubation in a MPs/H_2_O_2_/Cl^-^–containing medium. This endothelial cell injury was reduced in the presence of NaN_3_, taurine, or MPO inhibitor-I, while no injury was observed in HUVEC incubated with HBSS or MPs alone. As was also shown by others, H_2_O_2_ alone caused injury to HUVEC [23]. However, such H_2_O_2_-mediated damage was not inhibited by NaN_3_ or taurine (Figure 4). The fact that HUVEC were protected H_2_O_2_ damage when MPO inhibitors and neutrophil MPs were present suggests that other mechanism of H_2_O_2_ degradation (catalases, for instance) may also be associated with neutrophil MPs [24].

These findings highlight the participation of neutrophil MPs in cell injury and strongly suggest that these lesions preferably occur through a MPs/H_2_O_2_/Cl^-^ system-dependent mechanism. Besides the loss of membrane integrity, we observed morphological changes in endothelial cells after 2 and 16 minutes of incubation in MPs/H_2_O_2_/Cl^-^ system–containing medium. These changes included reductions in cell volume and the appearance of surface blebs. This toxicity diminished by pretreatment with taurine, a HOCl scavenger, indicating that this oxidant was involved in the process.

Finally, although the cell damage has been observed in other studies with purified HOCl, this is an original work because we showed that neutrophils MPs are capable of inducing endothelial cell injury by means of HOCl, a product generated by activated MPO, in presence of H_2_O_2_ and chlorine. No previous studies have shown that human vascular endothelial cells present with loss of membrane integrity and surface blebs when exposed to MPs/H_2_O_2_/Cl^-^ system. Hence, our results support the hypothesis that the generation of HOCl through a MPs-associated MPO/H2O2/Cl^-^–dependent mechanism may extend the endothelial damage induced by activated neutrophils, contributing to the severity of inflammatory diseases such as vasculitis and sepsis. It also would be interesting to study if similar mechanism of neutrophil mediated lesion is also involved in the damage of other cell types such as epithelial cells in ulcerous disease.

## Conclusion

MPs released upon neutrophil activation are capable of causing injury to vascular endothelial cells in an MPO-dependent manner. Hence, the MPs-associated MPO - hydrogen peroxide - chloride system may contribute to widespread endothelial cell damage in conditions of neutrophil activation as observed in vasculitis and sepsis.

## Abbreviations

AnV: Annexin V; CFDA: Carboxyfluorescein diacetate; CL: Chemiluminescence; DAPI: 4,6′-diamino-2-phenylindole; ECGS: Endothelial cell growth supplement; H_2_O_2_: Hydrogen peroxide; HBSS: Hank’s Balanced Salt Solution; HOCl: Hypochlorous acid; HUVEC: Human umbilical vein endothelial cells; LPS: Lipopolysaccharide; MPO: Myeloperoxidase; MPs: Microparticles; NaN_3_: Sodium azide; PBS: Phosphate-buffered saline; PI: Propidium iodide; PMA: Phorbol 12-myristate 13-acetate; PMN: Polymorphonuclear; PS: Phosphatidylserine; ROS: Reactive oxygen species; SEM: Scanning electron microscopy; TEM: Transmission electron microscopy.

## Competing interests

The authors declare that they have no competing interests.

## Authors’ contributions

TNP, LCPC, AAND, WLCS: Study design; TNP: Directly participated in the performance of all the experiments; TM and VCJR: Setting up and performance of some chemiluminescence experiments; LAF: Analysis of flow cytometry data; VMB and MSG: support on endothelial cell injury studies; WLCS was TNP supervisor during the whole work. All authors read and approved the final manuscript.
